# Inhibiting Mitochondrial Transcription to Model Alzheimer's Disease

**DOI:** 10.1002/alz70855_105994

**Published:** 2025-12-24

**Authors:** Anysja Roberts, Lesya Novikova, Ian Weidling, Xiaowan Wang, Alexander Paul Gabrielli, Colton R Lysaker, Heather M Wilkins, Russell H Swerdlow

**Affiliations:** ^1^ University of Kansas Alzheimer's Disease Research Center, Fairway, KS, USA; ^2^ University of Kansas Medical Center, Kansas City, KS, USA

## Abstract

**Background:**

Alzheimer's is a disease of aging and is currently defined as the accumulation of amyloid beta in the brain. During aging our mitochondrial function declines and this can lead to mitochondrial dysfunction. Accordingly, the mitochondria cascade hypothesis was developed and suggests mitochondria drive the characteristics found in Alzheimer's Disease (AD). The present research is focused on developing mitochondrial dysfunction models that can be used to model and study AD. This will enable us to determine if the decline in mitochondrial function results in AD hallmarks such as amyloid beta accumulation in the brain.

**Method:**

SH‐SY5Y cells and iPSC derived neurons were treated with 0.5 mM IMT1 to block mitochondrial transcription. SY5Y cells underwent treatment for 5‐days and neurons for 2‐weeks. Additionally, vehicle control groups were maintained for both cell lines. Mitochondrial DNA (mtDNA) copy numbers were measured with digital droplet PCR and mitochondrial RNA levels were measured with reverse transcription PCR. Western blots were used to measure changes in protein levels. Finally, seahorse assays were used to conduct mitochondrial stress tests and measure cellular oxygen consumption rates (OCR).

**Result:**

Following treatment, SY5Y mtDNA levels were significantly reduced, with mt‐TL1 DNA levels declining by 95% and D‐Loop DNA levels by 90%. Mt‐ND1 RNA levels were also impacted with a 90% reduction in SY5Y cells and 68% reduction in neurons. Additionally, mt‐CO2 protein levels were reduced by 81% in treated neurons. Finally, mitochondrial function was significantly reduced in treated SY5Y cells as indicated by a 64% decrease in mitochondrial OCR levels.

**Conclusion:**

Our findings indicate IMT1 treatment successfully reduces mitochondrial transcription as revealed by lower mt‐RNA and protein levels. Additionally, SY5Y treatment resulted in lower mtDNA copy numbers, aligning with lower mtDNA levels found in AD patients. Further, the decline in SY5Y mitochondrial activity suggests IMT1 induces mitochondria dysfunction. These findings indicate our model initiates mitochondrial dysfunction while demonstrating AD like characteristics, enabling us to use IMT1 treatment to model and study AD. Additional studies are ongoing to further characterize DNA, RNA, protein, enzymatic, and metabolic changes in both cell lines.

